# Supplementary material for: An open-source framework for large-scale, flexible evaluation of biomedical text mining systems
Source: J Biomed Discov Collab. 2008 Jan 29;3:1. doi: 10.1186/1747-5333-3-1 (PMC2276192; doi:10.1186/1747-5333-3-1)
Supplement: Additional file 1 — Evaluation platform source code. The additional file contains the software evaluation framework discussed in this paper. The most current release can be downloaded from SourceForge.net [1]. See the accompanying README file for instructions on installation and use. Also included in the distribution is a collection of UIMA wrappers for some commonly used BioNLP tools and annotated corpora. [file 1747-5333-3-1-S1.zip › bionlp_uima_repository-1.0.1/docs/api/src-html/edu/uchsc/ccp/uima/annotation/CCPAnnotationSet.html]

```
001    
002    
003    /* First created by JCasGen Tue Aug 28 16:44:10 MDT 2007 */
004    package edu.uchsc.ccp.uima.annotation;
005    
006    import org.apache.uima.jcas.JCas;
007    import org.apache.uima.jcas.JCasRegistry;
008    import org.apache.uima.jcas.cas.TOP;
009    import org.apache.uima.jcas.cas.TOP_Type;
010    
011    
012    /** The annotation set provides a means for arbitrarily categorizing or clustering groups of annotations. Annotations can be associated with multiple annotation groups. Examples of use include, defining Gold Standard annotation sets, and delineating between the use of different parameters during annotation, among others. Each annotation set is associated with a unique ID, a name and a description.
013     * Updated by JCasGen Tue Aug 28 16:44:10 MDT 2007
014     * XML source: /home/williamb/eclipse/RELEASED_SOURCE_CODE/descriptors/typeSystem/CCPTypeSystem.xml
015     * @generated */
016    public class CCPAnnotationSet extends TOP {
017      /** @generated
018       * @ordered 
019       */
020      public final static int typeIndexID = JCasRegistry.register(CCPAnnotationSet.class);
021      /** @generated
022       * @ordered 
023       */
024      public final static int type = typeIndexID;
025      /** @generated  */
026      public              int getTypeIndexID() {return typeIndexID;}
027     
028      /** Never called.  Disable default constructor
029       * @generated */
030      protected CCPAnnotationSet() {}
031        
032      /** Internal - constructor used by generator 
033       * @generated */
034      public CCPAnnotationSet(int addr, TOP_Type type) {
035        super(addr, type);
036        readObject();
037      }
038      
039      /** @generated */
040      public CCPAnnotationSet(JCas jcas) {
041        super(jcas);
042        readObject();   
043      } 
044    
045      /** <!-- begin-user-doc -->
046        * Write your own initialization here
047        * <!-- end-user-doc -->
048      @generated modifiable */
049      private void readObject() {}
050         
051     
052        
053      //*--------------*
054      //* Feature: annotationSetID
055    
056      /** getter for annotationSetID - gets An integer uniquely identifying a particular annotation set.
057       * @generated */
058      public int getAnnotationSetID() {
059        if (CCPAnnotationSet_Type.featOkTst && ((CCPAnnotationSet_Type)jcasType).casFeat_annotationSetID == null)
060          jcasType.jcas.throwFeatMissing("annotationSetID", "edu.uchsc.ccp.uima.annotation.CCPAnnotationSet");
061        return jcasType.ll_cas.ll_getIntValue(addr, ((CCPAnnotationSet_Type)jcasType).casFeatCode_annotationSetID);}
062        
063      /** setter for annotationSetID - sets An integer uniquely identifying a particular annotation set. 
064       * @generated */
065      public void setAnnotationSetID(int v) {
066        if (CCPAnnotationSet_Type.featOkTst && ((CCPAnnotationSet_Type)jcasType).casFeat_annotationSetID == null)
067          jcasType.jcas.throwFeatMissing("annotationSetID", "edu.uchsc.ccp.uima.annotation.CCPAnnotationSet");
068        jcasType.ll_cas.ll_setIntValue(addr, ((CCPAnnotationSet_Type)jcasType).casFeatCode_annotationSetID, v);}    
069       
070        
071      //*--------------*
072      //* Feature: annotationSetName
073    
074      /** getter for annotationSetName - gets The name of the annotation set.
075       * @generated */
076      public String getAnnotationSetName() {
077        if (CCPAnnotationSet_Type.featOkTst && ((CCPAnnotationSet_Type)jcasType).casFeat_annotationSetName == null)
078          jcasType.jcas.throwFeatMissing("annotationSetName", "edu.uchsc.ccp.uima.annotation.CCPAnnotationSet");
079        return jcasType.ll_cas.ll_getStringValue(addr, ((CCPAnnotationSet_Type)jcasType).casFeatCode_annotationSetName);}
080        
081      /** setter for annotationSetName - sets The name of the annotation set. 
082       * @generated */
083      public void setAnnotationSetName(String v) {
084        if (CCPAnnotationSet_Type.featOkTst && ((CCPAnnotationSet_Type)jcasType).casFeat_annotationSetName == null)
085          jcasType.jcas.throwFeatMissing("annotationSetName", "edu.uchsc.ccp.uima.annotation.CCPAnnotationSet");
086        jcasType.ll_cas.ll_setStringValue(addr, ((CCPAnnotationSet_Type)jcasType).casFeatCode_annotationSetName, v);}    
087       
088        
089      //*--------------*
090      //* Feature: annotationSetDescription
091    
092      /** getter for annotationSetDescription - gets A textual description of an annotation set.
093       * @generated */
094      public String getAnnotationSetDescription() {
095        if (CCPAnnotationSet_Type.featOkTst && ((CCPAnnotationSet_Type)jcasType).casFeat_annotationSetDescription == null)
096          jcasType.jcas.throwFeatMissing("annotationSetDescription", "edu.uchsc.ccp.uima.annotation.CCPAnnotationSet");
097        return jcasType.ll_cas.ll_getStringValue(addr, ((CCPAnnotationSet_Type)jcasType).casFeatCode_annotationSetDescription);}
098        
099      /** setter for annotationSetDescription - sets A textual description of an annotation set. 
100       * @generated */
101      public void setAnnotationSetDescription(String v) {
102        if (CCPAnnotationSet_Type.featOkTst && ((CCPAnnotationSet_Type)jcasType).casFeat_annotationSetDescription == null)
103          jcasType.jcas.throwFeatMissing("annotationSetDescription", "edu.uchsc.ccp.uima.annotation.CCPAnnotationSet");
104        jcasType.ll_cas.ll_setStringValue(addr, ((CCPAnnotationSet_Type)jcasType).casFeatCode_annotationSetDescription, v);}    
105      }
106    
107
```
